# Complimentary to the Albany Medical College

**Published:** 1870-09

**Authors:** 


### Complimentary to the Albany Medical College.

WE notice with surprise and disgust the following paragraph going the rounds of the Albany daily papers. If it was written by any of the well-wishers of the Institution, we think the Faculty might properly unite in prayer that God would " save them from their friends:"—

" THE WORLD MOVES.

LIBERALITY OF THE ALBANY MEDICAL SCHOOL—FRATERNIZATION OF ALLOPATHIC AND HOMŒOPATHIC PHYSICIANS.

The Hon. Ira Harris delivered the opening address at the college yesterday morning. The address was noble and interesting, and we were pleased to see many homœopathic physicians in attendance. Mr. Harris is a firm believer and patron of homœopathy, and fills a chair in the college. It is indeed gratifying to know that the barriers which have hitherto divided the two schools of medicine are being removed, and to see our college taking the initiatory step towards such a desirable achievement. We believe this is the only allopathic medical institution in this country that possesses views sufficiently liberal to allow any of the chairs to be filled by men who firmly and practically believe in the homœopathic doctrine. It is also pleasant to know that several of the trustees of the college are firm believers in homœopathy."

The Albany Medical College has been " kept before the people" for the past year to a remarkable degree; its history has been recorded, its struggles published, and its beauties shown with wood-cut illustrations, so that nothing can be more obvious than that its attractions have been presented to the popular view, but we cannot suppose that facts would warrant the above scandalous and disgraceful libel upon its previous good name and character.

Professors Quackenbush, Vanderpool and Mosier have resigned their professorships in this college, it is said, partly on account of conditions imprudently expressed in this newspaper item. If it is true that the Albany Medical College is in part homœopathic, and that such worthy and honorable men as those who recently resigned connection with it, did so from unwillingness to associate with, or in any way encourage such quackery, then we hope the next issues of the daily press will announce the general facts, and if they please, illustrate with original device, the union of rational medicine with the absurd and exploded dogmas of homœopathy.

